# A full year of turbulence measurements from a drift campaign in the Arctic Ocean 2019–2020

**DOI:** 10.1038/s41597-022-01574-1

**Published:** 2022-08-03

**Authors:** Kirstin Schulz, Volker Mohrholz, Ilker Fer, Markus Janout, Mario Hoppmann, Janin Schaffer, Zoé Koenig

**Affiliations:** 1 Oden Institute for Computational Engineering and Sciences, The University of Texas at Austin, Austin, TX USA; 2 Alfred Wegener Institute Helmholtz Centre for Polar and Marine Research, Physical Oceanography of Polar Seas, Bremerhaven, D-27570 Germany; 3 Leibniz Institute for Baltic Sea Research, Physical Oceanography and Instrumentation, Rostock-Warnemuende, D-18119 Germany; 4 Geophysical Institute, University of Bergen and Bjerknes Centre for Climate Research, Bergen, Norway; 5 Norwegian Polar Institute, Tromsø, Norway

**Keywords:** Physical oceanography, Fluid dynamics

## Abstract

Ocean turbulent mixing is a key process in the global climate system, regulating ocean circulation and the uptake and redistribution of heat, carbon, nutrients, oxygen and other tracers. In polar oceans, turbulent heat transport additionally affects the sea ice mass balance. Due to the inaccessibility of polar regions, direct observations of turbulent mixing are sparse in the Arctic Ocean. During the year-long drift expedition “Multidisciplinary drifting Observatory for the Study of Arctic Climate” (MOSAiC) from September 2019 to September 2020, we obtained an unprecedented data set of vertical profiles of turbulent dissipation rate and water column properties, including oxygen concentration and fluorescence. Nearly 1,700 profiles, covering the upper ocean down to approximately 400 m, were collected in sets of 3 or more consecutive profiles every day, and complemented with several intensive sampling periods. This data set allows for the systematic assessment of upper ocean mixing in the Arctic, and the quantification of turbulent heat and nutrient fluxes, and can help to better constrain turbulence parameterizations in ocean circulation models.

## Abstract (summary)

**Table:** 

Measurement(s)	temperature of water • conductivity of water • vertical shear • fluorescence of water • oxygen
Technology Type(s)	temperature sensor • conductivity sensor • shear probes • flurometer • oxygen sensor
Factor Type(s)	location and time
Sample Characteristic - Environment	ocean
Sample Characteristic - Location	Arctic Ocean

## Background & Summary

Sea ice cover and strong upper ocean stratification suppress vertical mixing in the Arctic Ocean, especially in the deep basins^1,2^. As the ice cover diminishes, momentum transfer from the atmosphere to the ocean, and the resulting turbulent mixing are expected to increase^2–5^. A large reservoir of heat and nutrients, originating from the Atlantic, resides at intermediate water depths. Primary production, and consequently food availability in the Arctic Ocean, is limited by nutrient supply, governed by physical transport processes^6,7^. Vertical mixing is hence a key process that will determine the fate of Arctic marine ecosystems^8,9^. In turn, increased primary production will amplify carbon dioxide fixation and drawdown. Moreover, increased heat fluxes in the ocean are an additional driver for sea ice loss, especially in winter and in regions where warm waters are located close to the surface^10^. Oceanic heat plays a leading role in the reduction of winter sea ice e.g. in the Barents Sea^11,12^ and increasingly further into the Arctic Ocean^13^. With longer ice-free seasons, more frequent turbulent mixing events above the continental shelf break regions might also change particle transport pathways and enhance vertical nutrient and heat transport in the Arctic^14^. A thorough knowledge and quantification of the Arctic Ocean mixing regime is hence crucial to understand the Arctic as a coupled system.

Direct observations of turbulent mixing in the central Arctic Ocean are still scarce, especially in the winter season. The first basin-scale survey of direct turbulence observations was conducted during the Beringia expedition in summer 2005, spanning the region from the Alaska continental shelf, crossing the North Pole to the Amundsen Basin^15^. Microstructure profiles were also obtained in the central Amundsen Basin e.g. in April 2007^1,16^, and April and August 2008^17^. Relatively more observations are available above the continental slopes north of Spitsbergen and above the Yermak Plateau (e.g. from summer 2007^18^, during the extensive N-ICE campaign from January to June 2015^10^, and from summer 2018^19^), as well as from the Laptev and East Siberian Sea (e.g. in summer 2007^20^, summer 2008^21^, and summer 2018^4^). In the Canada Basin, microstructure profiles have been obtained e.g. above the Chukchi slope in September 2015^22^, and in the central basin in August 2012^23^. Previous findings indicate that the Arctic Ocean is characterized by low levels of turbulence, compared to the other world’s oceans. Strong turbulent mixing is generally confined to the more energetic continental slope regions, either driven by tidal forces^2,19^ or episodic current surges^14^. However, previous measurements are strongly biased towards the summer season, and up to now, no data set covering a whole annual cycle of upper ocean turbulence exists.

Here, we present a comprehensive data set of vertical profiles of turbulent dissipation rates and vertically high-resolved profiles of temperature, salinity, oxygen, fluorescence and turbidity, measured with a tethered microstructure profiler in the upper ~350 m below the sea ice. Data were collected during the “Multidisciplinary drifting Observatory for the Study of Arctic Climate” (MOSAiC) drift campaign in the Arctic Ocean. The aim of this international expedition was to sample a whole annual cycle of the coupled Arctic system, using the icebreaker *RV Polarstern* as a drifting platform frozen into the sea ice. The experiment started in September 2019 in the Amundsen Basin, drifting northwards with the first ice floe^24^ parallel to the Lomonosov Ridge (legs 1 and 2, see Fig. 2a). In spring 2020, the drift speed accelerated and the floe passed the Gakkel Ridge into the Nansen Basin (leg 3). The expedition was interrupted in May/June 2020 due to the Covid-19 pandemic, when *Polarstern* had to leave the sampling floe to exchange personal near Svalbard. Upon return, measurements were resumed on the same floe at the northeastern flank of the Yermak Plateau (leg 4). After crossing the plateau, this first floe broke apart in Fram Strait at the end of July. Sampling continued on a second floe close to the North Pole after a short transit period back into the ice (leg 5, see Fig. 2). Except for the two sampling gaps between legs 3/4, and 4/5, profiles were obtained on a near-daily basis. Details on the course of the expedition can be found in overview publications^25–27^.

## Methods

### Sampling

#### Data acquisition

**Fig. 1:**
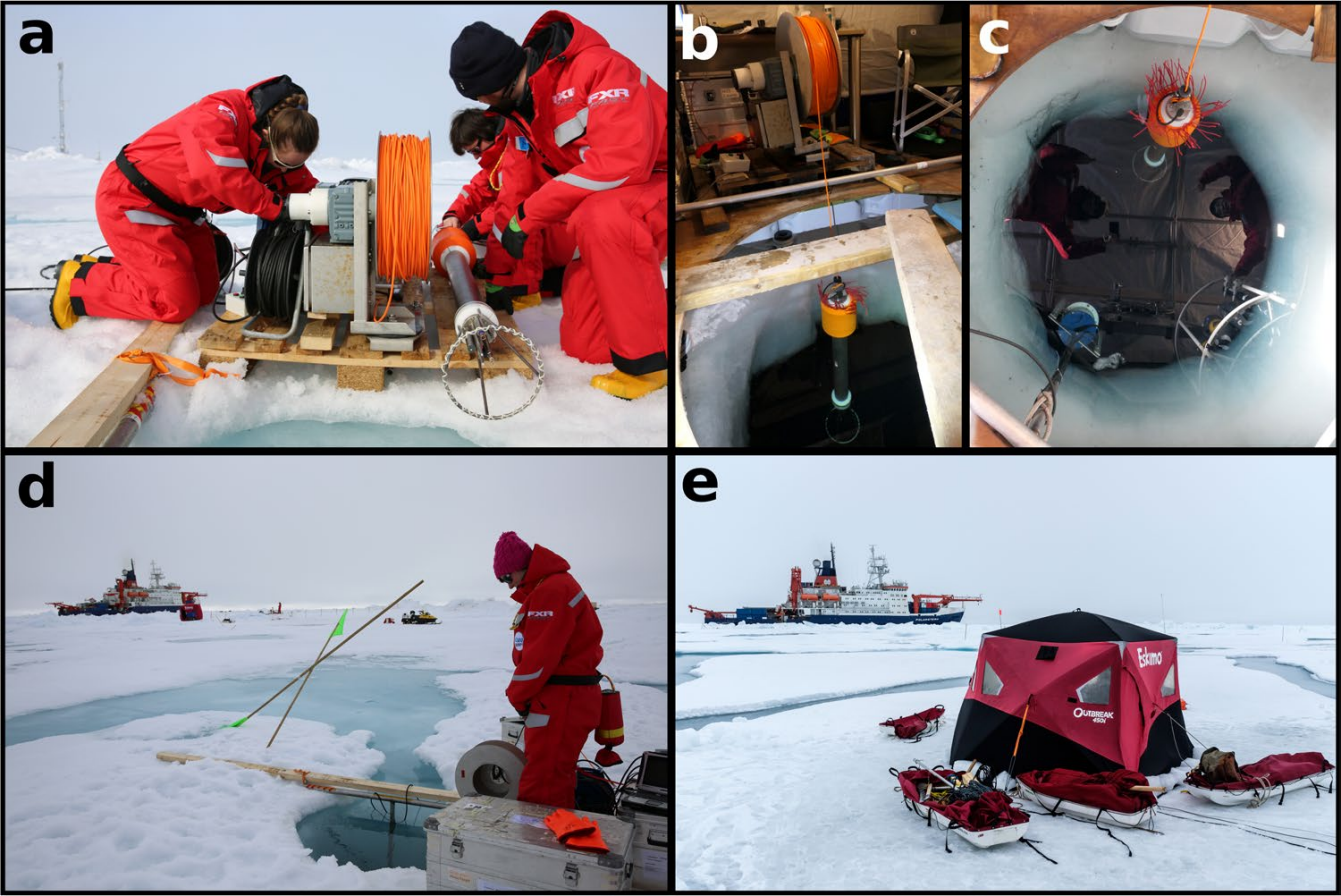
(**a**) MSS winch and profiler with sensors and protection cage (ⒸLisa Grosfeld), MSS setup during (**b**), (**c**) legs 1–3 within heated tent, (**d**) leg 4 (prior to installation of tent, with adjacent meltpond draining into hydrohole, ⒸMorven Muilwijk) and (**e**) leg 5.

Vertical profiles were collected using tethered, free-falling microstructure probes (MSS90L, Sea & Sun Technology, Germany), through a hole drilled in the sea ice, at a minimum distance of 250 m from *RV Polarstern*, to avoid disturbances generated by the ship’s keel (Fig. 1). Maps of the relative position of sampling sites on the floe, with “Ocean City” being the microstructure sampling site, can be found in the overview publications^25–27^. The probe was operated with an electrical winch installed at the edge of the ice hole. Power was provided via power lines from *RV Polarstern*, and from generators during times when the power lines had to be disconnected to prevent damage during dynamic ice conditions. Real-time data was received and stored on an acquisition laptop connected to the winch. During legs 1 to 3 (September 2019 to May 2020), sampling was performed within a heated tent to avoid sensor damage by freezing sea water (Fig. 1b,c). After the camp was re-established on legs 4 and 5, milder temperatures allowed for sampling from an unheated pop-up tent to protect the surface electronics from precipitation (Fig. 1e). Three different probes were used throughout the drift, equipped with different auxiliary sensors to sample biogeochemical parameters (see Table 1). The probes were free-falling with an approximate sinking velocity of 0.6 m s^−1^. Disturbances caused by cable tension (vibrations) and ice drift were minimized by feeding sufficient slack cable.

**Table 1:** Specification and sensors of the individual probes used during the MOSAiC drift.

	MSS046	MSS055	MSS091
Profile ID	0001–0833	4003–4274	8001–8221
			9001–9358
Sampling rate	1024 Hz	1024 Hz	512 Hz
Pressure	PA7-50	PA7-100; PA7-25	PA7-100
Shear 1	PNS 53	PNS 18	PNS C6100
Shear 2	PNS 54	PNS 19	PNS C6098
Temperature	PT100, NTC FP07	PT100, NTC FP07	PT100, NTC FP07
Conductivity	SST small	SST small	SST small
Acceleration	ADXL 203	ADXL 203	ADXL 203
Chlorophyll-a	—	—	Cyclops7 fluorometer
Oxygen	—	Fast SST-DO	SST-DO
Turbidity	—	SEAPOINT-Turb	—
Calibration date	June 2019 (≤167)	October 2018	March 2020 ( ≤ 8201)
	November 2020 (≥168)		Jan/Feb 2021 ( ≥ 8202)

Microstructure measurements were complemented with time and georeference data channels using a USB 2.0 u-blox 8 Multi GNSS receiver (NAVILOCK NL-8022MU) connected to the control computer. In 59 out of 1684 profiles, GPS data were missing for various reasons (broken connection, unavailable receiver). For these profiles, starting positions were taken from the GPS receiver of the 75 kHz ADCP installed in less than 300 m distance, which sampled position data at a temporal resolution of 1 minute. For the last station, which was performed after the ADCP was recovered, ship GPS data was used.

Sampling was carried out typically every day, comprising at least 3 and often more subsequent vertical profiles to obtain reliable daily averages. The regular schedule was complemented by 7 intensive sampling periods, with repeated profiling for more than 12 hours (see Table 3). Profiles usually covered the upper ocean between 2 m below the surface to depths between 300 to 450 m, depending on drift speed and cable length. Gaps in the time series were caused by a long interruption of the drift during the exchange between legs 3 and 4 (May 17 to June 19, 2020), and during the transit north after the floe broke up in Fram Strait (July 30 to August 22, 2020).

The sampling strategy summarized above had to be adapted to logistical and environmental conditions and cross-disciplinary coordinated efforts throughout the year. Some of the key events that directly influenced our measurements are outlined below, providing additional information that helps to interpret the data set. Interruptions of a few days due to ice movement or power loss are not included here. Further information can be found in the overview publication^25^ and the cruise reports.

- On November 16, 2019, high wind speeds around ~20 m s^−1^, resulting from a low-pressure system passing the region on November 16, caused the opening of an approximately 20 m-wide lead close to Ocean City the day after. Microstructure sampling operations were continued during the course of the wind event, providing first-hand observations of the ice-covered upper ocean under the influence of strong winds and in the proximity of a newly forming lead, e.g. a deepening of the surface mixed layer. The lead remained open for approximately one day. Throughout the course of the next days, the ice conditions changed and the previously separated parts of the floe started to converge. A massive pressure ridge started to form close by, constantly growing and moving towards Ocean City.
- On November 24, Ocean City had to be relocated to a safer site approximately 50 m away from the initial location.
- When regular sampling started after the drift interruption on June 27, the adjacent melt ponds had started to drain into the hydrohole used for microstructure operations. Draining had started sometime between June 22 and 25. As a consequence, the first 3–4 m of the water column consisted of a freshwater lens near freezing point (visible in Fig. 4b). The presence of this layer lead to false bottom formation at the base of the hydrohole. In the following weeks, the freshwater layer at the sampling location became successively shallower, until it could not be detected anymore in the microstructure profiles.
- After the floe breakup and relocation northward in August, the new hydrohole was located about 300 m away from the ship in an area of 1.3 m-thick level ice. The first location chosen was about 350 m away from the ship, but due to a crack in the logistic area, Polarstern had to relocate closer to the microstructure sampling spot.
- On August 22, 2020, the first sampling day on the second floe back north in the central Arctic Ocean, the camp had not been set up yet and profiles were performed in a pond that had melted through.
- During the first two out of three ice stations performed during the homebound transit at the end of leg 5, microstructure measurements were performed in leads about 400 m away from the ship.

**Fig. 2:**
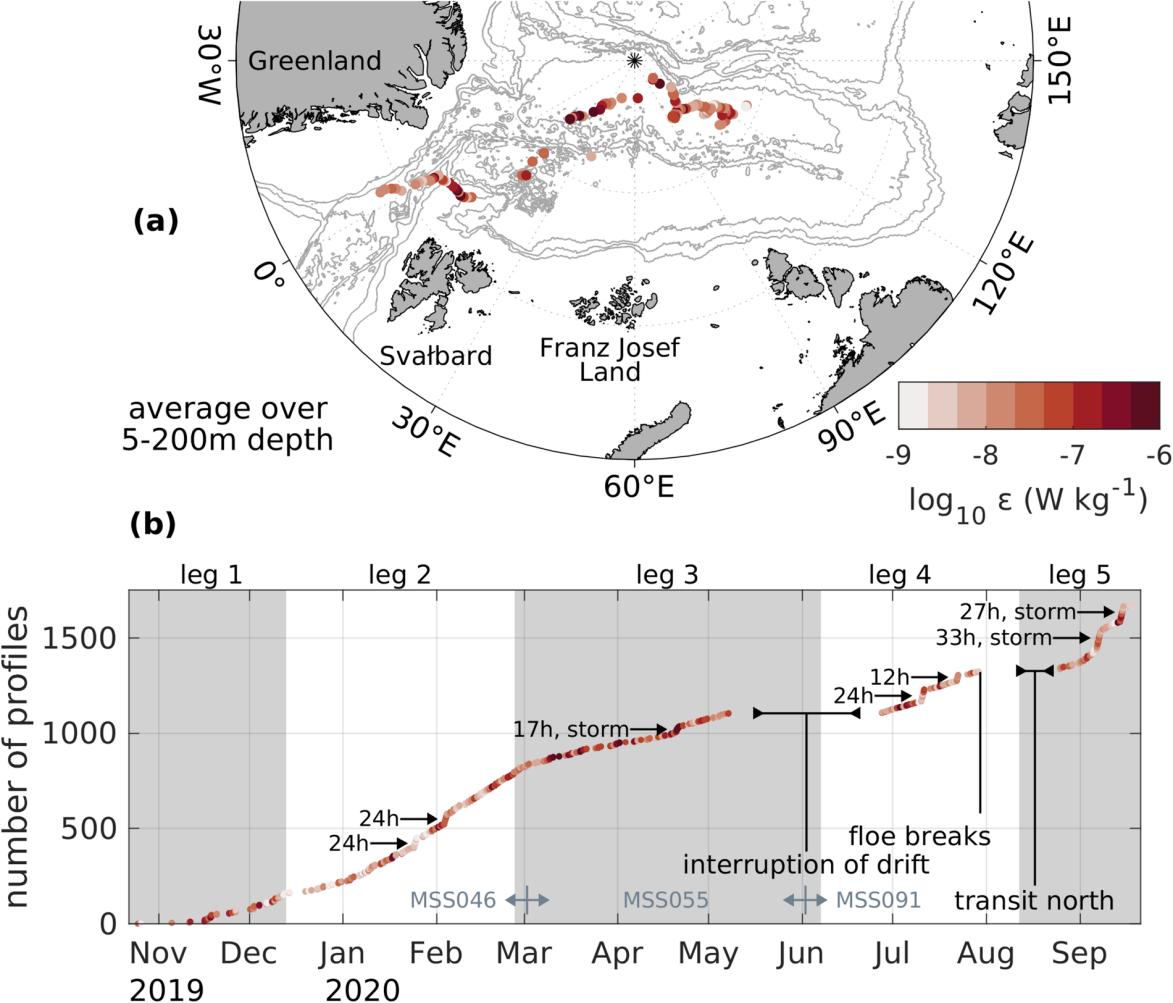
(**a**) Map of the Arctic Ocean, with gray lines denoting isobars with a spacing of 1000 m, and daily averaged sampling positions. Colors refer to the depth-averaged (5–200 m) dissipation rates. Bathymetric data was taken from the IBCAO data set^44^. (**b**) Time series of the cumulative number of individual profiles, with intensive sampling periods (see Table 3) and interruptions indicated. Colors refer again to the depth-averaged dissipation rates. Figure 2a was produced using the m_map matlab toolbox^45^.
- On May 7, 2020, data transmission failed after 3 profiles. The issue was caused by damage of the cable connecting the winch and the deck unit, and could only fixed in the beginning of leg 4 in June. Hence, no measurements were carried out in between.
- After the interruption of the drift, MSS055 was replaced by the new MSS091, to also measure chlorophyll fluorescence for bloom dynamics investigations.
- On July 23, 2020, the pressure sensor of MSS091 failed. It was replaced with the pressure sensor of the malfunctioning MSS075, but another test on July 25 showed that the temperature sensor was also not working properly. The temperature sensor was replaced with the one from MSS075, and a second test profile on July 25 was performed, showing reasonable data in most data channels, except for issues with the tilt and oxygen sensors, which delivered only constant voltage output after the sensor change.
- On August 29, 2020, the communication with the probe was interrupted and the deck unit lost power while the profiler was pulled up. Internal damage of the cable at about 100 m cable length from the termination was fixed by cutting and re-terminating the cable. The shorter cable restricted the depth range of the subsequent profiles to 250 m until the end of the expedition.
- On September 9, 2020, the instrument was opened and cable connections were cleaned, which resolved the issue of constant voltage output for the oxygen sensor. However, this sensor gave constant readings again in later profiles, probably due to corrosion or lose electrical connections. Hence, no oxygen data is available for profiles 8202–9232, 9341, and 9346–9358, and no tilt in x-direction for profiles 8202–9358.
- From 15 September, the communication between probe and data acquisition exhibited intermittent failures, likely due to a problem within the winch itself. Consequently, the cable was lowered manually, which was sometimes preventing the probe from free falling.

In addition to the environmental factors summarized above, the sampling was subject to several technical issues. A major problem was a low frequency (0.5 Hz) signal superimposed on most of the data channels of the profiler we initially planned to deploy (MSS075). This delayed the start of a regular sampling routine at the beginning of the expedition, and all attempts to fix this issue, including the replacement of an electronic board, were not successful. The profiler was not used, except for 15 test profiles in legs 1 and 2, which are not included in the published data set due to their questionable data quality. In addition, we encountered the following technical issues:

- The NTC temperature sensor of the MSS046 produced a cut-off for temperatures below −0.285 °C, probably caused by a sensor output that exceeded the range of the A/D converter of the probe. On December 17, 2019, this issue was fixed by replacing the NTC sensor. However, all temperature measurements taken with the probe before that day are affected. For these first profiles (1–167) the pre-cruise manufacturer calibration was applied.
- On January 1, 2020, the connector of the data cable of the MSS winch was repaired and fixed, after same data transmission errors during the measurements.
- On February 5, 2020, the cable termination of the MSS winch was repaired after occasionally failures of the SDA data aquisition software.
- On March 2, 2020, MSS046 was replaced by MSS055, to also measure biogeochemical parameters (oxygen, turbidity) before the beginning of the spring bloom (Fig. 2b).

### Data processing

Microstructure data are processed using a Matlab toolbox developed at the Institute for Baltic Sea Research, in collaboration with the instrument manufacturer (see section “Code Availability”). Processing routines use the Matlab Signal Processing Toolbox and the GSW Oceanographic Toolbox of TEOS-10^28^.

#### Data conversion and initial quality control

Binary raw data files are read in and converted to physical units, using the latest available manufacturer calibration (see Table 1). Data transmission errors are identified as gaps in the count sensor. The missing data lines are added and filled with NaN values. To account for the different relative position of the individual sensors on the profiler head with respect to the vertical sinking direction, the time series of all sensors are aligned with the level of the shear sensors, assuming a constant sinking velocity of 0.6 m s^−1^. To reduce salinity spikes, an e-folding filter is applied to the conductivity data record that adjusts the time constant of the sensor to that of the PT100 temperature sensor. In addition, visually identified sections with bad data quality in individual sensors are either linearly interpolated (e.g. spikes in conductivity) or set to NaN values (e.g. longer periods of sensor malfunction). The probes hardware includes a 1 Hz electronic high pass filter, applied directly before the sensor signal is preamplified in the sensor shaft electronics. Hence, no further high-pass or anti-aliasing filter is applied in the data processing.

The upper bound of the vertical profile is set to 2.1 m, since the low vertical velocity at the beginning of the profiles often caused a mismatch in the sensor alignment or disturbed measurements above this depth. To identify the lower bound of the profile, the maximum pressure, the vertical velocity of the probe, and the signal of acceleration sensor are used. The pressure profile is smoothed by applying a running mean (1 m vertical distance window size) and a low-pass filter (fourth order Butterworth) with a cut-off period of 1 second. The vertical sinking velocity is calculated as the time derivative (using the constant sampling frequency) of the smoothed and filtered pressure record. The lower bound of each profile is then identified as the shallowest depth within the lowermost 10% of the profile where the probe’s sinking velocity falls below 0.3 m s^−1^. The vertical velocity is then re-calculated by smoothing the original, cut-off record with two running means (2.3 seconds and 1 second window), and calculating the time derivative (using the constant sampling frequency).

For each shear sensor, the raw data (*sh*_*raw*_) is converted to a voltage (*V*) according to

$$V=4.577707\times 1{0}^{-5}+9.155423\times 1{0}^{-5}\times s{h}_{raw}.$$

Data points in the shear records above 3 m depth (where the probe is typically not perfectly free-falling) and at low (<0.3 m s^−1^) sinking speeds, which indicate tension on the cable, are discarded. Vertical shear (*sh*) is calculated from the time derivative ∂_*t*_*V* as

$$sh=\frac{{c}_{0}{\partial }_{t}V}{{s}_{t}{v}^{2}},$$

where ${c}_{0}={\left(2\sqrt{2}\Omega {\rho }_{0}\right)}^{-1}$ is a constant including the probe-specific electronic gain of the shear sensor, Ω = 11, and the mean sea water density *ρ*_0_ = 1024 kg m^−3^. The derivative ∂_*t*_*V* is obtained using a one (left) sided gradient, calculated from two subsequent data points. This shifts the gradient profile by 0.5 ms (or 0.25 mm) relative to the other data channels. Since the higher noise of this approach is in the high frequency range of the spectrum that is not used for estimating dissipation rates (see section below), the impact on the results is small. The respective sensitivity of the individual shear sensors *s*_*t*_ is calculated dependent on the *in-situ* temperature *T* as ${s}_{t}={s}_{t}^{* }\left({c}_{0}+{c}_{1}T+{c}_{2}{T}^{2}+{c}_{3}{T}^{3}\right)$, using temperature-dependant post-cruise calibrations (see Table 2). All meta data and raw data channels are summarized in Tables 4, 5, respectively.

**Table 2:** Calibration coefficients of the deployed shear sensors (sensitivity ${s}_{t}^{* }$ determined at reference temperature T = 20 °C).

Sensor	Date	${s}_{t}^{* }$	*c*_*0*_	*c*_1_	*c*_2_	*c*_3_
PNS 18	Nov 24, 2021	0.0004799	0.77243	0.0039163	0.00050035	−6.3624 × 10^−6^
PNS 19	Nov 10, 2020	0.0005778	0.72894	0.018065	−0.00062125	1.9782 × 10^−5^
PNS 53	Nov 12, 2020	0.0006361	0.82652	−0.00018223	0.00078041	−1.6879 × 10^−5^
PNS 54	Nov 13, 2020	0.0007402	0.79861	0.0026669	0.00035976	5.1918 × 10^−7^
PNS C6100	Oct 21, 2021	0.0005875	0.86894	0.0016275	0.00018186	3.2202 × 10^−6^
PNS C6098	Oct 21, 2021	0.0006924	0.80615	−0.00031938	0.00076336	−1.3138 × 10^−5^

**Table 3:** List of extensive sampling periods.

Time (UTC)	Profile IDs	Comment
Jan 24, 10:15 – Jan 25, 11:30	0398–0454	diurnal sampling
Feb 03, 10:15 – Feb 04, 11:30	0521–0578	diurnal sampling
Apr 20, 06:30 – Apr 20, 23:30	4175–4201	storm event
Jul 10, 09:45 – Jul 11, 09:45	8061–8125	coordinated diurnal sampling activity
Jul 22, 02:45 – Jul 22, 15:00	8168–8201	coordinated with team ATMOS
Sep 06, 09:30 – Sep 07, 18:15	9112–9209	storm event, coordinated with team ECO
Sep 14, 04:45 – Sep 15, 08:15	9258–9340	storm event, coordinated with team ECO

**Table 4:** Summary of the metadata included in the data set.

Parameter	Short name	Symbol	Unit	Short description
Start time	stime	—	date (UTC)	start date and time from external GPS
Start latitude	slat	—	decimal degree	start latitude from external GPS
Start longitude	slon	—	decimal degree	start longitude from external GPS
Time	gpstime	—	date (UTC)	date and time from external GPS
Latitude	lat	—	decimal degree	latitude from external GPS
Longitude	lon	—	decimal degree	longitude from external GPS
PC time	rtctime	—	date (UTC)	date and time from control PC
Time elapsed	time	t	seconds	since start of profile

For some profiles, no external GPS data is available, time and location at the start of profiling were substituted using other sources.

**Table 5:** Summary of raw data channels included in the data set.

Parameter	Short name	Symbol	Unit	Short description
Pressure	press	p	dbar	PA7-100 pressure sensor
Pressure	p250	—	dbar	PA7-25 pressure sensor (more accurate at shallow depths)
*In-situ* temperature	temp	T	°C	PT100 temperature sensor
Conductivity	cond	—	mS cm^−1^	conductivity sensor
*In-situ* temperature	ntc	—	°C	NTC temperature sensor (fast responding)
Probe acceleration	acc	—	m s^−2^	ADXL 203 acceleration sensor
Probe tilt x-axis	accx	—	decimal degree	ADXL 203 acceleration sensor
Probe tilt y-axis	accy	—	decimal degree	ADXL 203 acceleration sensor
Dissolved oxygen (raw)	rawo2	—	mV	SST-DO oxygen sensor
Turbidity	turb	—	FTU	SST-Turb optical backscatter turbidity sensor
Fluorescence	chl-a	—	—	Cyclops7 fluorometer

Some parameters are not available for individual probes, see Table 1.

#### Dissipation rate estimates

Under the assumption of isotropic turbulence, i.e. that statistical turbulence properties are independent of direction, the rate of turbulent kinetic energy dissipation by viscous forces *ε* is related to the shear variance

$$\varepsilon =15\nu \overline{{\left(\frac{\partial u}{\partial z}\right)}^{2}},$$

where *ν* is the kinematic molecular viscosity, $$\frac{\partial u}{\partial z}$$ is the vertical shear, and the overbar denotes a spatial average^29^. For seawater, the viscosity is temperature dependent and is approximated as $$\nu =(1.792747-0.05126103T+$$$$0.0005918645{T}^{2})\times 1{0}^{-6}\;{{\rm{m}}}^{2}\;{{\rm{s}}}^{-1}$$ ^30^.

The shear variance is often estimated by integrating the shear spectrum over a finite wave number space. Here, we use the empirical Nasmyth spectrum^31^, an accepted reference shear spectrum that can be approximated in non-dimensional form^32^ as

$$\Psi =\frac{8.05{x}^{\frac{1}{3}}}{1+{(20.6x)}^{3.715}},$$

where *x* = *kL*_*k*_ is the (cyclic) wavenumber *k*, normalized by the Kolmogorov length scale ${L}_{k}={\left(\frac{{\nu }^{3}}{\varepsilon }\right)}^{0.25}$.

To calculate dissipation rates from the shear spectrum, each shear time series is subdivided into segments of 1 second, with an overlap of 0.5 seconds, and the linear trend per section is removed. A Bartlett window and a fast Fourier transformation are applied, and the one-sided power spectrum is calculated. Treating both shear sensors independently, all observed spectra within one vertical bin (here: 1 m) are averaged into a mean spectrum, that is iteratively fitted to the Nasmyth spectrum. Starting with a theoretical spectrum corresponding to a dissipation rate of *ε* = 10^−7^ W kg^−1^, at each iterative step the dissipation rate estimate is corrected according to the cumulative error of the fit, until this error is smaller than 0.01 or a maximum number of 50 iterative steps is reached. Working in cyclic units, the wavenumber range used for the fit is 2 cpm to the minimum of 30 cpm or 0.4 *L*_*k*_. The reduction factor of 0.4 for the upper limit when using the Kolmogorov length scale is applied as the measured spectra do not follow the theoretical shape close to the edge of the inertial subrange for low turbulent dissipation rates. The final dissipation rate *ε* is then calculated as the mean of the two estimates from the two shear probes.

The deviation of the spectral fit, i.e. the root mean square of the deviation from the measured spectrum and the Nasmyth spectrum in the used wavenumber range, is saved as quality control parameter (see Table 6). This deviation is generally smaller (<0.1) at high dissipation rates (>10^−8.5^ W kg^−1^), and up to 0.3–0.4 at low dissipation rates.

**Table 6:** Summary of derived parameters included in the data set.

Parameter	Short name	Symbol	Unit	Short description
Vertical velocity of the probe	vvel	—	m s^−1^	from smoothed pressure profile
Practical salinity	psal	S	—	TEOS-10^28^
Absolute salinity	asal	*S*_*A*_	g kg^−1^	TEOS-10^28^
Potential temperature	ptemp	*θ*	°C	TEOS-10^28^
Conservative temperature	ctemp	Θ	°C	TEOS-10^28^
Potential density anomaly	sigma0	*σ*_*θ*_	kg m^−3^	TEOS-10^28^
*In-situ* density	rho	*ρ*	kg m^−3^	TEOS-10^28^
Brunt-Väisälä frequency	bvf2	N^2^	s^−2^	TEOS-10^28^
Turner angle	Tu ang	*Tu*	decimal degree	TEOS-10^28^
Density stability	R rho	*R*_*ρ*_	—	TEOS-10^28^
Thorpe scale	thorpe sc	—	m	^33^
Thorpe displacement	thorpe dis	—	m	^33^
Sorted density anomaly	sigma0 sort	—	kg m^−3^	^33^
*In-situ* oxygen saturation	oxy s	—	%	^36^
Oxygen concentration	oxy c	—	*μ*mol kg^−1^	^37^
Dissipation rate	epsilon1	—	log_10_(W kg^−1^)	dissipation rate from shear sensor 1 (logarithmic)
Quality parameter	epsq1	—	—	standard deviation of spectral fit, shear sensor 1
Dissipation rate	epsilon2	—	log_10_(W kg^−1^)	dissipation rate from shear sensor 2 (logarithmic)
Quality parameter	epsq2	—	—	standard deviation of spectral fit, shear sensor 2
Dissipation rate	epsilon	*ε*	log_10_(W kg^−1^)	dissipation rate of turbulent kinetic energy (logarithmic)
Pseudo dissipation rate	peps	—	log_10_(W kg^−1^)	dissipation rate calculated from internal acceleration sensor for noise level estimates (logarithmic)
Covariance peps, eps1	covar1	—	—	covariance between pseudo dissipation rate and dissipation rate from shear sensor 1
Covariance peps, eps2	covar2	—	—	covariance between pseudo dissipation rate and dissipation rate from shear sensor 2
Diffusion coefficient	krho	*K*_*ρ*_	log_10_(m^2^ s^−1^)	${K}_{p}=0.2\frac{\varepsilon }{{N}^{2}}$^34^
Buoyancy Reynolds number	Re b	*Re*_*b*_	—	$R{e}_{b}=\frac{\varepsilon }{\nu {N}^{2}},$ (kinematic molecular viscosity *ν*).
Ozmidov length scale	L oz	*L*_*oz*_	m	${L}_{oz}=\sqrt{\frac{\varepsilon }{{N}^{3}}}$
Diffusion coefficient	krho s	*K*_*ρ*_	log_10_(m^2^ s^−1^)	${K}_{\rho }=2\nu \sqrt{R{e}_{b}}$^35^

Some parameters are not available for individual probes, see Table 1.

Dissipation rate values are set to NaN if one of the following conditions is met: 1. Dissipation rate estimates for individual sensors are unrealistically low (*ε* < 10^−13^ W kg^−1^); 2. The covariance between shear from the individual sensors and the probe acceleration (calculated in the time domain) exceeds the threshold of 0.2; or 3. Dissipation rate estimates from the individual sensor deviate by more than a factor of 5.

Another quality control parameter included in the data set is the pseudo dissipation rate, calculated from the pseudo shear, i.e. the ratio between probe acceleration and sinking velocity, with the acceleration measured by a shear probe installed inside the pressure case of the profiler. The pseudo shear is treated identical to the physical shear, giving an estimate of pseudo dissipation rate, which is a proxy for the noise caused by the vibrations of the probe itself, and should be much smaller than the dissipation rate estimates.

#### Derived quantities

Based on measured *in-situ* temperature and conductivity, practical salinity, absolute salinity, potential and conservative Temperature, *in-situ* density, potential density anomaly (with sea surface as reference), the Brunt-Väisälä frequency, and the Turner angle and stability ratio were calculated using the TEOS-10^28^ set of equations (see Table 6). Thorpe length scales and displacements^33^ were calculated using the potential density profiles. Thorpe displacements are obtained relative to the statically stable, sorted density profile, using a noise level of 0.01 kg m^−3^.

Additional parameters included in the data set are the diffusion coefficient ${K}_{\rho }=\Gamma \frac{\varepsilon }{{N}^{2}}$, using Γ = 0.2^34^, the buoyancy Reynolds number $R{e}_{b}=\frac{\varepsilon }{\nu {N}^{2}}$, the Ozmidov length scale ${L}_{oz}=\sqrt{\frac{\varepsilon }{{N}^{3}}}$, and an alternative estimate of the diffusion coefficient calculated as ${K}_{\rho }=2\nu \sqrt{R{e}_{b}}$^35^.

Turbidity, fluorescence and oxygen data are only processed using the manufacturer calibration and were not compared against *in-situ* measurements. Oxygen saturation is calculated as the ratio between the manufacturer-calibrated raw oxygen data and the partial oxygen pressure at 100% saturation $p10{0}_{{O}_{2}}$, where $p10{0}_{{O}_{2}}=0.2095({p}_{atm}-{p}_{{H}_{2}O})$, with *p*_*atm*_ = 1013.25 mbar being the standard air pressure at sea level and ${p}_{{H}_{2}O}=6.112{\rm{\exp }}(\frac{17.62}{(243.12+T)})$ being the saturated water vapor pressure at *in-situ* temperature T^36^. To calculate the oxygen concentration (in *μ*mol kg^−1^), the oxygen saturation is multiplied with the theoretical oxygen concentration at 100% saturation derived from the *in-situ* temperature and density and the practical salinity^37^. A complete list of all derived parameters is given in Table 6.

## Data Records

Data and meta data of all microstructure profiles are provided in a single netCDF file, with a vertical resolution of 1 m^38^. In addition, the corresponding raw data files are provided in three data sets, sorted by the individual probe used^39–41^. All data sets are published on PANGAEA (https://www.pangaea.de). The data is under a moratorium and will be publicly available as of January 1, 2023 in accordance with the MOSAiC data policy.

## Technical Validation

**Fig. 3:**
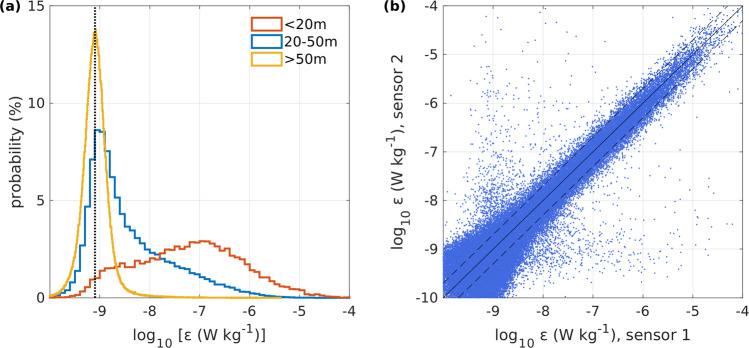
(**a**) Distribution of dissipation rate in the upper 20 m (red), 20–50 m (blue) and lower than 50 m (yellow). The vertical dashed line indicates 8 × 10^−10^ W kg^−1^ (**b**) Scatter plot of the dissipation rate estimates from both shear sensors. Dashed lines indicate a deviation of ± 2.

The relative distribution of measured dissipation rates within different depth intervals is displayed in Fig. 3a. In the energetic surface layer, above 20 m depth, dissipation rates highly depend on the surface forcing, e.g. the ice drift speed, and range from 10^−9^ to 10^−6^ W kg^−1^. In the intermediate layer at 20–50 m depth, turbulence appears to follow the classic log-normal distribution. In deeper quiescent layers, distribution of dissipation rates accumulate near a peak at 8 × 10^−10^ (vertical dotted line in Fig. 3a), suggesting a lower detection level, i.e. noise level, for dissipation estimates of about 10^−9^ W kg^−1^.

A comparison between dissipation rate estimates from the two independent shear sensors show a generally good agreement within a factor of 2 (dashed lines in Fig. 3b), especially at dissipation rates greater than 10^−8^ W kg^−1^. At lower dissipation rates, the agreement between both sensors decreases. Overall, our quality control indicates that the presented dissipation rate estimates are robust in the presence of elevated turbulence at values greater than 10^−8^ W kg^−1^. Dissipation rate estimates at lower turbulence levels are more uncertain.

The accuracy of the auxiliary sensors given by the manufacturer are ±0.01 °C (NTC FP07); ±0.002 °C (PT100); ±0.002 mS cm^−1^ (conductivity). The microstructure data has not been compared with measurements from any other CTD (conductivity, temperature, depth) system that was deployed during the MOSAiC drift yet. MSS046 and MSS055 were mounted on the CTD frame and lowered together with the CTD in Ocean City (full sensor package) down to 200 m depth on January 30 (MSS046) and February 7 (MSS055). A calibration cast with a stand-alone SST 48 M CTD (serial no. 1459; Sea & Sun Technology, Germany, only pressure, temperature and salinity) attached to the MSS091 was carried out on July 29, 2020. Especially when using the auxiliary data, i.e. fluorescence, turbidity, and oxygen, we recommend to perform a cross-calibration, either using the calibration casts or several casts closest in time, to obtain quantitatively reliable data.

## Usage Notes

From the presented dissipation rate measurements and vertical diffusion coefficients, turbulent fluxes of e.g. heat can be calculated. In combination with additional data sets obtained during the MOSAiC campaign, e.g. vertical profiles of nutrient concentration or the sea ice mass balance, the effect of turbulent mixing on nutrient supply and the energy balance can be assessed, providing insights into the Arctic system beyond the field of physical oceanography.

**Fig. 4:**
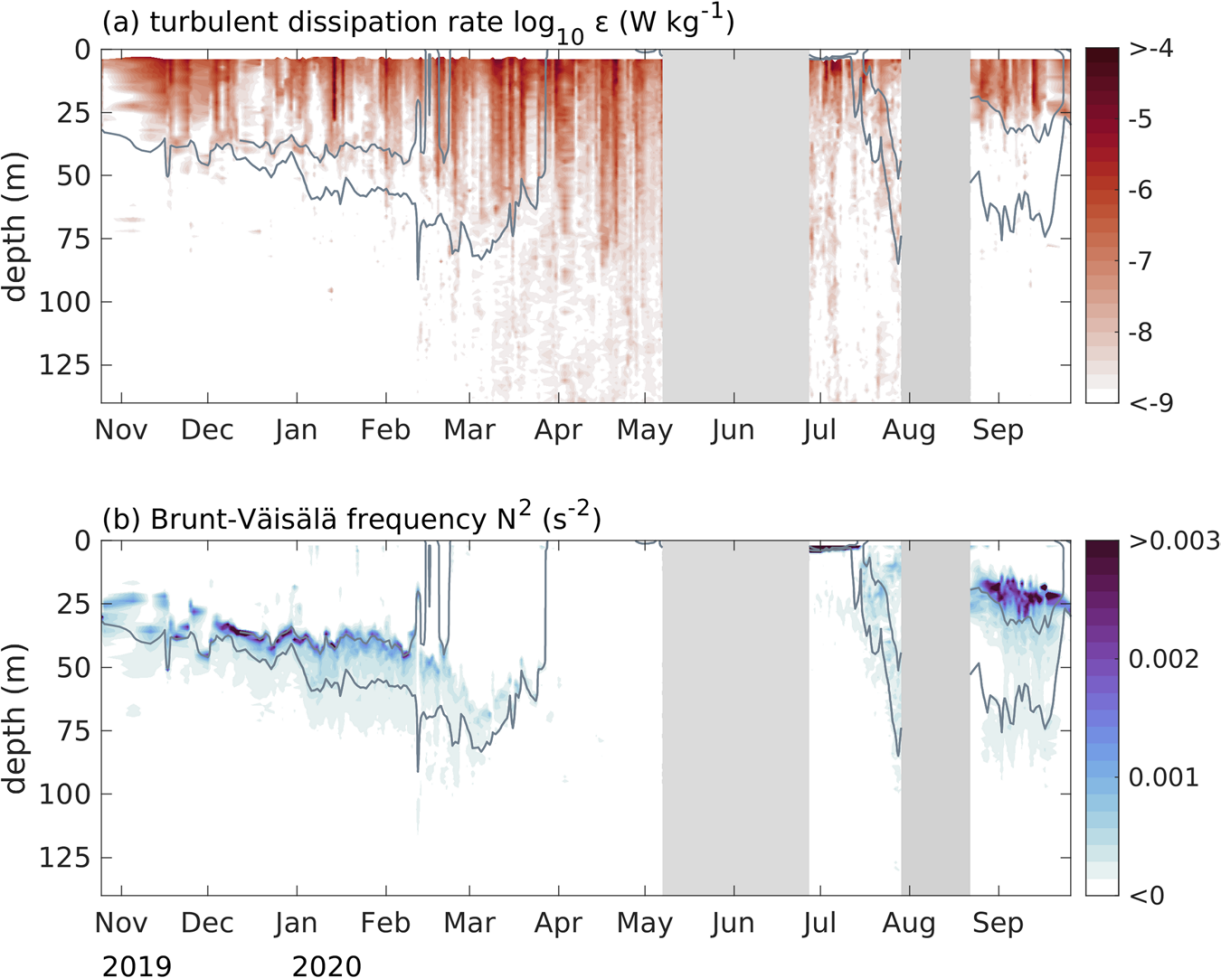
Daily averaged (**a**) turbulent dissipation rate (W kg^−1^), and (**b**) Brunt-Väisälä frequency (s^−2^) in the upper 140 m. Gray isolines indicate potential density anomaly of *σ*_*θ*_ = 26, 27, 29, 30, 31 kg m^−3^, gray patches indicate the two major interruptions of the drift.

To illustrate the possibilities for scientific research this data set provides, two key quantities, the turbulent dissipation rate *ε* and the water column stratification *N*^2^, are displayed in Fig. 4. From September to February 2019, strong turbulent dissipation rates are confined to the near surface layer. During the course of the drift, we observed a successive deepening of the mixed layer from November to March, along with a reduction of upper ocean stratification arising from a successive increase in near surface salinity. This evolution is probably attributed to ongoing ice growth and associated brine rejection, but might partly reflect spatial gradients along the drift pathway away from the large Siberian freshwater sources. The erosion of the upper ocean stratification allowed for a deeper penetration of the surface enhanced turbulence, starting in March (Fig. 4a). In June, the presence of a fresh melt water layer appears as a strong stratification in the upper meters of the water column, and we observed an increase in upper (<75 m) ocean stratification starting mid-July. During this part of the drift, enhanced turbulent dissipation rates at greater depth are probably related to the complex topography at Yermak Plateau. After the relocation north, enhanced turbulence is again mostly confined to the upper 30 m of the water column, the surface mixed layer is considerably shallower and the upper ocean stratification is stronger compared to the first half of the drift.

## Data Availability

The code used for data processing is available with the data set at the data repository. A publication of the processing routines on a maintained public repository is in preparation.
